# ADELLE: A global testing method for Trans-eQTL mapping

**DOI:** 10.1101/2024.02.24.581871

**Published:** 2024-02-26

**Authors:** Takintayo Akinbiyi, Mary Sara McPeek, Mark Abney

**Affiliations:** 1 Department of Statistics, The University of Chicago, Chicago, IL, US; 2 Department of Human Genetics, The University of Chicago, Chicago, IL, US

## Abstract

Understanding the genetic regulatory mechanisms of gene expression is a challenging and ongoing problem. Genetic variants that are associated with expression levels are readily identified when they are proximal to the gene (i.e., cis-eQTLs), but SNPs distant from the gene whose expression levels they are associated with (i.e., trans-eQTLs) have been much more difficult to discover, even though they account for a majority of the heritability in gene expression levels. A major impediment to the identification of more trans-eQTLs is the lack of statistical methods that are powerful enough to overcome the obstacles of small effect sizes and large multiple testing burden of trans-eQTL mapping. Here, we propose ADELLE, a powerful statistical testing framework that requires only summary statistics and is designed to be most sensitive to SNPs that are associated with multiple gene expression levels, a characteristic of many trans-eQTLs. In simulations, we show that ADELLE is more powerful than other methods at detecting SNPs that are associated with 0.2–2% of the traits. We apply ADELLE to a mouse advanced intercross line data set and show its ability to find trans-eQTLs that were not significant under a standard analysis. This demonstrates that ADELLE is a powerful tool at uncovering trans regulators of genetic expression.

## Introduction

eQTL mapping, in which association is tested between gene expression levels and genetic variants, is a useful approach toward understanding mechanisms of genetic regulation. Cis-eQTLs, genetic variants that influence expression of proximal genes, are often readily detected because their effect sizes are commonly large, and the local nature of their effects limits the number of tests and, hence, the multiple testing burden. Because of this, many studies have focused on investigating the role of cis-regulatory effects on gene expression. Recent work, however, has estimated that cis-genetic effects account for a minority of human complex trait variance, perhaps as little as 11%, while trans-genetic effects, i.e. causes that are distant from the gene being regulated, may account for 70% or more of complex trait variance in humans [1,2]. Unfortunately, even though trans-eQTL effects may dominate the genetic variability of gene expression and of complex traits, the identification of trans-eQTLs has been impeded by two significant hurdles. Compared to cis-eQTLs, trans-eQTLs are much harder to detect because their effect sizes tend to be smaller [2], and the space of possible genes whose expression they might be associated with is much bigger, leading to a higher burden of multiple comparisons.

A basic approach in both model organisms and humans to detect trans-eQTLs is to perform, for each SNP, a test of association against every trans-gene [1,3–5]. To account for multiple testing, either a Bonferroni correction is applied or a false discovery rate (FDR) procedure is used. Because of the very high number of tests performed, only the strongest of signals achieve statistical significance. This has led to recent efforts to develop methods that will be more effective at detecting trans-eQTLs. Broadly, many of the methods seek to increase the number of discoveries by applying at least one of the following strategies (1) reducing the multiple testing burden by either reducing the number of variants tested [6–10] or reducing the number of genes tested [11–13], or (2) leveraging the expectation that a trans-eQTL will influence the regulation of multiple genes [12–15]. Although incorporating biological or other external information to effectively make the number of tests smaller has the potential to increase power by eliminating either variants or traits where the null hypothesis is true, it also has the potential to miss important signals. On the other hand, even though a trans-eQTL may affect the expression levels of multiple genes the number of these genes will typically be a very small fraction of the total number of genes. Together, these qualities have made the development of effective tools for the discovery of trans-eQTLs very challenging.

We address the problem of developing a powerful statistical method for trans-eQTL detection. In particular, we frame the problem as one where we seek to reject the global null hypothesis that for a candidate trans-eQTL (e.g., a single SNP) none of the expression traits are associated with the SNP. We develop a method that requires only summary statistics of individual tests of association between a SNP and an expression trait. Advantages of only requiring summary statistics include their ease of being shared and savings in the person and computational effort to generate them.

For the general statistical problem of aggregating a collection of Z scores or p-values into a single test of the global null hypothesis, various methods have been proposed. Examples include Simes’s method [16], higher criticism [17,18], the Berk-Jones statistic [19], and methods based on equal local levels (ELL) [19–24]. Both the higher criticism and Berk-Jones statistics have generalizations to the case where the tests are dependent, generalized higher criticism [25] and generalized Berk-Jones [26]. These methods were used to test association between a SNP-set and an outcome. Another class of global tests commonly used in genetics corresponds to the sum of $\chi^{2}$ statistics from different tests [27] and generalizations of that, e.g., SKAT [28] and other variance component tests [29]. The CPMA [14] method has been proposed for combining test statistics for multi-trait mapping.

In general, there is no uniformly most powerful test of the global null hypothesis. Instead, different tests will be optimal in different alternative model regimes. For instance, the min-p test, with a multiple testing correction, should do well when there is at least one large Z score. On the other hand, sums of $\chi^{2}$ types of tests (e.g. SKAT) are likely to do well when weak signals are spread over a relatively large proportion of the Z scores. Here, we propose ADELLE, which is an extension of ELL to the case of dependent tests. Because ADELLE is an ELL-based test, we expect it to show strong performance when the signal is both relatively weak and sparse within a collection of Z scores, which is the situation we expect when searching for trans-eQTLs. We assess the performance of ADELLE relative to other methods through simulation studies and application to trans-eQTL detection in mouse data from an advanced intercross line [4].

### Description of the method

We first briefly consider the simplified case in which the expression traits are assumed to be independent and describe how the ELL global testing method could be applied. Then we describe ADELLE, our extension of ELL to the case of dependent traits, which we apply to trans-eQTL mapping.

### Global trans-eQTL testing with ELL

In an eQTL mapping study in which $\tilde{D}$ expression traits and M genome-wide SNPs are observed on each of N individuals, suppose each expression trait is tested for association with each genome-wide SNP in the sample leading to a $\tilde{D}\times M$ summary statistic matrix Π of p-values having (d,m)th entry $\pi_{dm}$ equal to the p-value for testing association between expression trait d and SNP m in the N individuals. In this subsection we make the simplifying assumption that the $\tilde{D}$ traits are independent. We extend to the case of dependent traits in the following subsection.

For a given SNP m, define ${𝒟}_{m}$ to be the subset of expression traits that are considered trans to it, from among the larger set of $\tilde{D}$ traits measured. To detect trans-eQTLs, we propose to perform M global hypothesis tests, one for each SNP, in which the mth global hypothesis test has null and alternative hypotheses

(1) $$H_{0}^{\left(m\right)}\;:\;\text{SNP}\;m\;\text{is}\;\text{not}\;\text{associated}\;\text{with}\;\text{any}\;\text{trait}\;\text{in}\;{𝒟}_{m}$$

(2) $$H_{A}^{\left(m\right)}\;:\;\text{SNP}\;m\;\text{is}\;\text{associated}\;\text{with}\;\text{at}\;\text{least}\;\text{one}\;\text{trait}\;\text{in}\;{𝒟}_{m}.$$

We now fix a SNP m and describe the ELL method for performing the mth global hypothesis test, where the test statistic is constructed from the p-values in column m of Π. Specifically, we consider a vector of p-values π of length $D_{m}=\left|{𝒟}_{m}\right|$, consisting of the subset of p-values in the mth column of Π that correspond to the traits in ${𝒟}_{m}$. For simplicity of exposition, we drop the subscript m in the remainder of this subsection, so we consider 𝒟 to be the set of traits that are trans to the SNP and consider π to be of length D. Under the null hypothesis that the given SNP is not associated with any of its trans traits and the further assumption of independence of traits (and assuming that the method for calculating p-values is well-calibrated), the entries of π would be D i.i.d. Uniform(0,1) random variables.

ELL is a general global testing method that models the entries of π as i.i.d. from a distribution having cumulative distribution function (cdf) $F_{\pi }(x)$ for x∈(0,1). The null hypothesis would be

(3) $$H_{0}\;:F_{\pi }(x)=x\;\text{for}\;\text{all}\;x\in (0,\;1)\text{,}$$

i.e., the p-values are Uniform(0,1), and the one-sided alternative hypothesis would be

(4) $$H_{A}\;:F_{\pi }(x)>x\;\text{for}\;\text{at}\;\text{least}\;\text{one}\;x\in (0,\;1)\text{,}$$

i.e., the p-values tend to be smaller under the alternative than would expected under the null. We use the notation $\pi =\left(\pi_{1},\ldots ,\pi_{D}\right)$ and for 1≤d≤D, we define $\pi_{\left(d\right)}$ to be the dth order statistic of π, i.e., we sort the entries of π in ascending order and let $\pi_{\left(d\right)}$ be the dth component of the sorted vector, so $\pi_{\left(1\right)}\leq \pi_{\left(2\right)}\leq \ldots \leq \pi_{\left(D\right)}$. Under the null hypothesis that the unsorted p-values $\pi_{1},\ldots ,\pi_{D}$ are i.i.d. uniform, the entries of $\left(\pi_{\left(1\right)},\ldots ,\pi_{\left(D\right)}\right)$ are dependent with a known joint distribution, and marginally each $\pi_{\left(d\right)}$ has the Beta(d,D−d+1) distribution for 1≤d≤D.

The ELL test starts by comparing each order statistic to its corresponding beta null distribution and deciding whether it is smaller than expected. Then the ELL test statistic is based on the order statistic that shows the most significant deviation from its corresponding null distribution. On the one hand, if trans-eQTL signals are only of moderate or weak size, then, e.g., $\pi_{\left(1\right)}$ and $\pi_{\left(2\right)}$ might actually represent null tests, and the true alternatives could be represented by smaller than expected $\pi_{\left(d\right)}$ for values of d that are perhaps of small to moderate size. On the other hand, finding that $\pi_{\left(d\right)}$ is smaller than expected only for larger values of d, e.g., d close to D, would be difficult to interpret and might not seem compelling evidence for the SNP being a trans-eQTL. Therefore, we propose to base the ELL test statistic on only the smallest fraction q of the p-values, i.e., on order statistics $\pi_{\left(d\right)}$ for 1≤d≤qD, where q∈(0,1). In the original formulation of ELL, Berk and Jones [19] used q=.5. In the eQTL mapping context, a smaller q would seem more appropriate, and we take q=.2, i.e., we only the consider the smallest 20% of the p-values for a given SNP. For simplicity of notation, in what follows we assume that qD turns out to be an integer (otherwise it could be replaced by floor(qD)).

To construct the ELL test statistic, we first calculate qD “l-values”, one for each $\pi_{\left(d\right)}$, 1≤d≤qD, where the l-value $l_{d}$ for $\pi_{\left(d\right)}$ is the p-value for testing the null hypothesis that $\pi_{\left(d\right)}$ is drawn from a Beta(d,D−d+1) distribution vs. a one-sided alternative for which we reject the null hypothesis if $\pi_{\left(d\right)}$ is sufficiently small. Thus, $l_{d}=pbeta\left(\pi_{\left(d\right)},d,D-d+1\right)$ where pbeta(x,a,b) is the cdf of the Beta(a,b) distribution evaluated at x. Then the ELL test statistic is

$$T_{ELL}=\underset{1\leq d\leq qD}{min}l_{d}.$$

To assess whether the SNP is a trans-eQTL, we perform a one-sided hypothesis test at level α based on $T_{ELL}$, where we reject the null hypothesis in Eq 1 if $T_{ELL}<\eta$, where η (the “local level”) is a function of α. We refer to this as an equal local level test because the local level η at which we reject $H_{0}$ is equal for all $l_{d}$. That is, if any of the l-values are less than η we reject $H_{0}$. Previous work [23] shows that the ELL test is asymptotically optimal for detecting deviations from a Gaussian distribution for a wide class of rare-weak contamination models.

For the case when the traits are independent, there are existing algorithms [24,30,31] to calculate the global level α of the test as a function of the local level η, where we call this function α(η). Most algorithms are for the case q=1, but could be adapted to other q. For example, if we let ξ=floor(qD), then α(η) could be obtained as $1-\sum_{j=0}^{\xi -1}c_{j}^{\left(\xi \right)}$, where $c_{j}^{\left(\xi \right)}$ is a quantity calculated recursively in Algorithm 1 of Appendix B.2 of Weine et al. [24] To invert the function α(η) and determine the local level η corresponding to a chosen global level α for the ELL test, a binary search can then be conducted to find the needed η.

### ADELLE: extension of the ELL method to dependent traits

The ELL approach described in the previous subsection assumes independence of traits, but in practice there is typically correlation among gene transcript levels. Our goal is still to perform, for each SNP, a global test based on the null and alternative hypotheses in Eq 3 and 4. However, dependence among traits leads to dependence among the elements of the p-value vector π. In that case, it is no longer true that, e.g., $\pi_{\left(d\right)}$ is beta distributed under the null as it is in the independence case. Therefore, the methods we describe above for calculation of the ELL test statistic and its null distribution are no longer applicable.

The ADELLE method we propose generalizes the ELL approach to allow for dependent traits. For 1≤d≤D, define $F_{\left(d\right)}$ to be the cdf of the distribution for $\pi_{\left(d\right)}$ under the null hypothesis in the case when the traits are dependent. The basic idea behind ADELLE is that we find an approximation to $F_{\left(d\right)}$ and use it to calculate the qD l-values $l_{1},\ldots ,l_{qD}$ in the case when the traits are dependent. Then we define the ADELLE test statistic $T_{ADELLE}$ to be the minimum of $\left\{l_{1},\ldots ,l_{qD}\right\}$. Finally, we calculate the p-value for the ADELLE test using a Monte Carlo approximation method given below.

First we describe how dependence is incorporated into the model. Rather than directly modeling the dependence on the p-value scale, we instead consider a set of association test statistics $Z_{1},\ldots Z_{D}$, where $Z_{d}$ tests association between the given SNP and its d th trans trait, 1≤d≤D. We assume that under the null hypothesis, each $Z_{d}∼N(0,\;1)$, where they can be correlated with each other, and we assume that $\pi_{d}$ is a two-sided p-value based on $Z_{d}$, i.e., $\pi_{d}=2\Phi \left(-\left|Z_{d}\right|\right)$, where Φ is the standard normal cdf.

Let G denote the genotype vector of the SNP and $Y_{d}$ the phenotype vector of its d th trans trait. Typical examples of $Z_{d}$ would be the t-statistic for testing significance of G in a linear model for $Y_{d}$ or the Wald t-statistic for testing significance of G in a linear mixed model (LMM) for $Y_{d}$. In large samples, such a t-statistic will be approximately standard normal under the null hypothesis or, if necessary, could be transformed to be approximately standard normal under the null hypothesis by applying the transformation $\Phi^{-1}\left(pt\left(Z_{d}\right)\right)$ where pt is the cdf of the t-distribution with degrees of freedom =N−k−1 where k is the number of predictors in addition to the intercept in the linear model or LMM. A likelihood ratio $\chi^{2}$ test statistic for testing significance of G in a LMM for $Y_{d}$ could also be converted to such a $Z_{d}$ value by taking a square root of the $\chi_{1}^{2}$ test statistic and applying the sign of the estimated coefficient of G in the LMM for $Y_{d}$.

We let $Z={\left(Z_{1},\ldots ,Z_{D}\right)}^{T}$ and, under the global null hypothesis that the SNP is unassociated with any of its trans traits, we model Z as multivariate normal:

(5) $$Z∼N_{D}(0,\;\Omega ),$$

where $N_{D}$ denotes the multivariate normal distribution of dimension D, 0 is a vector of 0 's of length D and Ω is a D×D correlation matrix. For the moment, we take Ω as known, but we describe below how to estimate it. To calculate $F_{\left(d\right)}(h)$ for h∈(0,1), where $F_{\left(d\right)}$ is the cdf of $\pi_{\left(d\right)}$ under the null hypothesis, we first point out the key identity that the two events $E_{1}=\{\pi_{\left(d\right)}\leq h\}$ and $E_{2}=\{\sum_{k=1}^{D}I\{\pi_{k}\leq h\}\geq d\}$ are the same, where I{⋅} is the indicator function that equals 1 if the event inside the brackets occurs and 0 otherwise, and where $E_{2}$ is saying that at least d of the p-values are ≤h. By the defined relationship between $\pi_{k}$ and $Z_{k}$, we have that the events $\left\{\pi_{k}\leq h\right\}$ and $\left\{\left|Z_{k}\right|\geq -\Phi^{-1}(h/2)\right\}$ are the same, so $E_{2}=\{\sum_{k=1}^{D}I\left\{\left|Z_{k}\right|\geq -\Phi^{-1}(h/2)\right\}\geq d\}$. Next, define $S(c)=\sum_{d=1}^{D}I\{\left|Z_{d}\right|\geq c\}$ for c≥0, where S(c) counts the number of $\left|Z_{d}\right|$ that are greater than or equal to c, and note that $E_{2}=\left\{S\left(-\Phi^{-1}(h/2)\geq d\right\}\right.$. Therefore, the following two events are the same

(6) $$\left\{\pi_{\left(d\right)}\leq h\right\}=\left\{S\left(-\Phi^{-1}(h/2)\right)\geq d\right\}.$$

Finally, we have for the l-value

(7) $$l_{d}(h)\equiv F_{\left(d\right)}(h)\equiv P_{0}\left(\pi_{\left(d\right)}\leq h\right)=P_{0}\left(S\left(-\Phi^{-1}(h/2)\right)\geq d\right),$$

where $P_{0}(\cdot )$ represents probability under the null hypothesis that the SNP is not associated with any of its D trans traits.

As a consequence, we can obtain needed values of $F_{\left(d\right)}$ by considering the distribution of S(c) under the null hypothesis. If Ω=I, then for c≥0, S(c) has the null distribution of a Binomial(D,2Φ(−c)) random variable. When Ω≠I, S(c) has the same null mean as a Binomial(D,2Φ(−c)), but the null variance of S(c) is strictly greater than that for Binomial(D,2Φ(−c)), i.e., the distribution of S(c) is over-dispersed relative to binomial. The beta-binomial distribution is a standard choice for modeling binomial-like data when there is over-dispersion. Therefore we approximate the distribution of S(c) with a beta-binomial distribution BB(D,λ,γ) where λ and γ are chosen so that the first and second moments match those of S(c), using techniques of a previous work [32] (see also [33]). The details are given in S1 Text. From the resulting approximation to the distribution of S(c), we obtain an approximation to $F_{\left(d\right)}$, which we call ${\overset{ˆ}{F}}_{\left(d\right)}$, based on Eq. 7.

The required calculation of ${\overset{ˆ}{F}}_{\left(1\right)}(h),\ldots ,{\overset{ˆ}{F}}_{\left(qD\right)}(h)$ for all h∈ℋ can be efficiently carried out as a pre-computation step, as described in detail in S1 Text.

To obtain the ADELLE test statistic, we first obtain the qD l-values $l_{1},\ldots ,l_{qD}$, where $l_{d}$ is defined to be ${\overset{ˆ}{F}}_{\left(d\right)}$ evaluated at the observed value of $\pi_{\left(d\right)}$. Then the ADELLE test statistic is given by $T_{ADELLE}={min}_{1\leq d\leq qD}l_{d}$. In the special case when Ω=I, we get back the same ELL l-values and ELL test statistic used for the independence case in the previous subsection.

### Assessment of significance of ADELLE

We use a Monte Carlo approach to assess significance of the ADELLE test statistic. Specifically, we simulate R i.i.d. vectors ${\tilde{Z}}^{\left(r\right)}∼N_{D}(0,\overset{ˆ}{\Omega })$, 1≤r≤R, where R is very large, and for each ${\tilde{Z}}^{\left(r\right)}$, we calculate the ADELLE statistic, call it $T^{\left(r\right)}$. For any observed ADELLE statistic, T, we calculate its p-value as (N(T)+1)/(R+1), where $N(T)=\sum_{r=1}^{R}I\{T^{\left(r\right)}\leq T\}$ counts the number of $T^{\left(r\right)}$ values that are less than or equal to T.

### Covariance matrix estimation

In an eQTL mapping study in which $\tilde{D}$ expression traits and M genome-wide SNPs are observed on each of N individuals, let Z denote the $\tilde{D}\times M$ matrix of test statistics, where $Z_{dm}$, the (d,m) th entry of Z, is the test statistic for association between trait d and SNP m, and where each $Z_{dm}$ is assumed to be standard normal under the null hypothesis of no association between trait d and SNP m. We further assume that N≪min(D,M), i.e., there are many more expression traits and SNPs than there are individuals in the study, and that rank(Z)=N−1 (or, more generally, rank(Z)=N−k−1 if k PCs, PEER factors, or other covariates have been regressed out of the expression traits in addition to an intercept). The low rank of Z occurs because there are only N individuals providing data for all $\tilde{D}*M$ tests. (See S1 Text for more details.)

For simplicity of exposition, we ignore for the moment the distinction between cis- and trans-eQTLs and assume that the global null hypothesis for each SNP is that it is not associated with any of the $\tilde{D}$ traits. Following that, we show how to extend the covariance matrix estimation method to trans-eQTL mapping specifically.

Under the null hypothesis that none of the SNPs are eQTLs for any of the traits, we assume that

(8) $$Z_{\cdot ,m}∼N_{\tilde{D}}\left(0,\Omega \right)holds\;for\;1\leq m\leq M,$$

where $Z_{\cdot ,m}$ is the m th column of Z, consisting of the tests of association of SNP m with each of the $\tilde{D}$ traits, and where Ω is a $\tilde{D}\times \tilde{D}$ correlation matrix of rank $\tilde{D}$ that we need to estimate. (See S1 Text for further details on the model for Z.) In the simple special case in which there is no population structure, there are no covariates, and Z is based on simple linear regression, then the model in Eq 8 can be shown to hold with Ω equal to the true correlation matrix for the $\tilde{D}$ traits (see S1 Text). More generally, Ω would involve other features of the model used to calculate Z, so we would need to use Z to estimate Ω, rather than estimating it directly from the trait data.

To estimate Ω, we consider a two-step strategy in which we first define a simple estimator ${\overset{ˆ}{\Omega }}_{1}$ to be the sample correlation matrix for the rows of Z, i.e., ${\overset{ˆ}{\Omega }}_{1}=cov2cor\left(Z\left(I_{M}-1_{M}1_{M}^{T}\right)Z^{T}\right)$, where cov2cor is the function that maps a symmetric positive semi-definite matrix A with positive diagonal elements to a matrix of the same size with (i,j) th element $A_{ij}/\sqrt{A_{ii}A_{jj}}$. Note that ${\overset{ˆ}{\Omega }}_{1}$ has a special structure that results, first, from the fact that N, the number of individuals in the study, is much less than $\tilde{D}$. With only N replicates of the $\tilde{D}$-dimensional trait vector available in the data, ${\overset{ˆ}{\Omega }}_{1}$, like Z, is effectively of rank N−1 (or, more generally, N−k−1) and is subject to “spread” of its N−k−1 top eigenvalues (see, e.g., [34] and [35]). There is an additional effect due to the fact that ${\overset{ˆ}{\Omega }}_{1}$ is formed based on Z values for M different SNPs which results in additional spread of its top eigenvalues, though this additional spread will be lessened due to the fact that M is large. As a result, it is necessary to perform regularization on ${\overset{ˆ}{\Omega }}_{1}$ to obtain a good final estimator $\overset{ˆ}{\Omega }$ of Ω.

To regularize ${\overset{ˆ}{\Omega }}_{1}$, we apply a form of eigenvalue shrinkage (see, e.g., [34] and [35]). Suppose ${\overset{ˆ}{\Omega }}_{1}=P\Lambda P^{T}$ is the eigendecomposition of ${\overset{ˆ}{\Omega }}_{1}$, where Λ is a diagonal matrix of eigenvalues that has dth diagonal element $\lambda_{d}$, where $\lambda_{1}\geq \lambda_{2}\geq \ldots \lambda_{\tilde{D}}\geq 0$. Since ${\overset{ˆ}{\Omega }}_{1}$ has 1's on the diagonal, we also have $\sum_{d=1}^{\tilde{D}}\lambda_{d}=\tilde{D}$. As a first step, we apply shrinkage to calculate a new set of eigenvalues ${\tilde{\lambda }}_{1}\geq {\tilde{\lambda }}_{2}\geq \ldots \geq {\tilde{\lambda }}_{\tilde{D}}\geq 0$, and a new diagonal matrix $\tilde{\Lambda }$ whose d th diagonal element is ${\tilde{\lambda }}_{d}$, as follows: for our setting in which the number of observations N is small relative to $\tilde{D}$ and M, we define $\gamma =dim({\overset{ˆ}{\Omega }}_{1})/rank({\overset{ˆ}{\Omega }}_{1})$ and let $\lambda_{+}=(1+\sqrt{\gamma }{)}^{2}$. Let $u=\left|\left\{d\;:\lambda_{d}>\lambda_{+}\right\}\right|$, and call $\left\{\lambda_{1},\ldots ,\lambda_{u}\right\}$ the "large" eigenvalues and $\left\{\lambda_{u+1},\ldots ,\lambda_{\tilde{D}}\right\}$ the "small" eigenvalues. We apply a debiasing function $f_{1}$ to each of the large eigenvalues and a linear contraction $f_{2}$ to each of the small eigenvalues. Here $f_{1}\left(\lambda_{d}\right)=\;.5(\lambda_{d}+1-\gamma +\sqrt{{\left(\lambda_{d}+1-\gamma \right)}^{2}-4\lambda_{d}})$, where $f_{1}$ is the inverse bias function for the large eigenvalues in a spiked covariance model for a case when ${\overset{ˆ}{\Omega }}_{1}$ is not low-rank [34]. For $f_{2}$, we use a linear contraction $f_{2}\left(\lambda_{d}\right)=a+b\lambda_{d}$, where a and b are chosen to satisfy the constraints that $f_{2}\left(\lambda_{+}\right)=f_{1}\left(\lambda_{+}\right)$, i.e., that $f_{1}$ and $f_{2}$ agree at the boundary between small and large eigenvalues, and that $\sum_{d=1}^{\tilde{D}}{\tilde{\lambda }}_{d}=\tilde{D}$. The resulting values of a and b, which satisfy a>0 and 0<b<1, are given in S1 Text. Given $\tilde{\Lambda }$, we obtain our final covariance matrix estimator $\overset{ˆ}{\Omega }$ as

(9) $$\overset{ˆ}{\Omega }=\mathrm{cov}2\mathrm{cor}\left(P\tilde{\Lambda }P^{T}\right).$$

To do trans-eQTL mapping, we must adjust for the fact that different SNPs may have different traits for which they are trans. As before, let ${𝒟}_{m}$ be the subset of the $\tilde{D}$ traits for which SNP m is trans, for 1≤m≤M, and let $D_{m}=\left|{𝒟}_{m}\right|$. Define $Z_{{𝒟}_{m},m}$ to be the $D_{m}\times 1$ sub-vector of $Z_{\cdot ,m}$ consisting of only those elements corresponding to traits in ${𝒟}_{m}$. Let $\Omega_{{𝒟}_{m}}$ be the $D_{m}\times D_{m}$ sub-matrix of Ω consisting only of those rows and columns corresponding to the traits in ${𝒟}_{m}.$ Then Eq 8 becomes

$$Z_{{𝒟}_{m},m}∼N_{D_{m}}\left(0,\Omega_{{𝒟}_{m}}\right)\;\text{for}\;1\leq m\leq M.$$

To form ${\overset{ˆ}{\Omega }}_{1}$, we first define ${\overset{ˆ}{V}}_{1}$, and then set ${\overset{ˆ}{\Omega }}_{1}=cov2cor({\overset{ˆ}{V}}_{1})$, where ${\overset{ˆ}{V}}_{1}$ has (i,j) th entry

$$\sum_{\left\{m:\{i,j\}\subset {𝒟}_{m}\right\}}\left(Z_{i,m}-{\overset{‾}{Z}}_{i}\right)\left(Z_{j,m}-{\overset{‾}{Z}}_{j}\right)$$

and

$${\overset{‾}{Z}}_{i}=c^{-1}\sum_{\left\{m:i\in {𝒟}_{m}\right\}}Z_{i,m},\;\text{where}\;c=\left|\left\{m\;:i\in {𝒟}_{m}\right\}\right|,$$

for $1\leq i\leq \tilde{D}$ and $1\leq j\leq \tilde{D}$. We then regularize to form $\overset{ˆ}{\Omega }$ as described.

### Identifying the expression traits associated with a significant trans-eQTL

When the null hypothesis of Eq 1 is rejected for a given SNP in favor of the alternative that the SNP is associated with at least one of the expression traits for which is it trans, it is obviously of interest to know for which traits the SNP is a trans-eQTL. We use the following method developed by Peterson et al. 2016 [36]. Let M be the total number of SNPs in the study that were tested by the ADELLE global testing method, and let m (possibly 0) be the number of those that were declared to be significant based on some genome-wide cutoff. Then for each SNP i that was declared significant by ADELLE, we take the set of p-values, $\pi_{di}$, $1\leq d\leq D_{m}$ for testing association between SNP i and each trait it is trans to, and apply FDR with target false discovery rate $\alpha \frac{m}{M}$, where we use α=0.05. The set of traits discovered by this method are the ones for which SNP i is determined to be a trans-eQTL. Peterson et al. 2016 [36] show that this method is effective at controlling the sFDR at level α, meaning that conditional on a given SNP being declared significant by a global testing method such as ADELLE, the false discovery rate for the traits it is associated with is effectively controlled at level α.

### Simulation methods

In the simulations, we consider a setting in which we have summary statistics from association tests of a SNP with each of $D=10^{4}$ expression traits, and we want to combine the summary statistics into a global test of the null hypothesis that the SNP is not associated with any of the traits. We use each of the different global testing methods described below to perform the global test. To assess type 1 error, we obtain an empiric null distribution by generating 10^4^ simulation replicates in which the SNP is not associated with any of the D traits and perform each of the global tests on each replicate, thereby obtaining the distribution of the statistic under the null hypothesis. We then use this distribution to obtain a p-value when performing the global test for a given Z vector. To compare power across methods, we generate 10^3^ simulation replicates in which the SNP is associated with exactly R of the D traits, where we perform studies for each of several choices of R from 10 to 200. The effect size of the SNP on each of the R associated traits is set to be $c_{R}$, where $c_{R}$ is chosen so that the maximum power across the methods is approximately in the range 0.5–0.9. We compare the power of the different methods based on the proportion of replicates in which each method rejects the null hypothesis.

In each simulation replicate, we simulate a vector of Z scores of length D from a multivariate normal distribution with mean vector μ=0 under the null hypothesis with a correlation matrix as described below. Under the alternative hypothesis, we simulate the Z scores from the same distribution as under the null hypothesis but where the mean vector has exactly R of the D entries equal to $c_{R}$ and the remaining D−R entries equal to 0. To simulate the process of estimating the correlation matrix Ω, it is important to capture the special structure of ${\overset{ˆ}{\Omega }}_{1}$, the D×D sample correlation matrix of Z that is used as the first step in the estimation of Ω. (The second step in the estimation is to regularize ${\overset{ˆ}{\Omega }}_{1}$ by performing eigenvalue shrinkage as described above.) In S1 Text, we show that in our simulation setting, conditional on Y, Z has the matrix normal distribution $Z|Y∼{MN}_{\tilde{D},M}(0,{\overset{ˆ}{C}}_{Y},I)$ under the null hypothesis, where ${\overset{ˆ}{C}}_{Y}$ is the D×D sample correlation matrix for the given Y values, and where, in the simulations, we assume independent SNPs for simplicity. This fact justifies the following procedure for simulating ${\overset{ˆ}{\Omega }}_{1}$: first we simulate N i.i.d. replicates of Y from $N_{D}(0,\Omega )$ and form a D×D sample correlation matrix ${\overset{ˆ}{C}}_{Y}$ from the replicates, where ${\overset{ˆ}{C}}_{Y}$ is of rank N−1. We choose N=208, which is the sample size in the mouse data set we analyze below, and we choose Ω to give a similar correlation matrix to expression trait correlation matrix observed in the mouse data set. Then we simulate Z by obtaining M i.i.d. replicates from $N_{D}(0,{\overset{ˆ}{C}}_{Y})$, and form the D×D sample correlation matrix ${\overset{ˆ}{\Omega }}_{1}$ from those. Then we perform the regularization procedure described above on ${\overset{ˆ}{\Omega }}_{1}$ to obtain $\overset{ˆ}{\Omega }$, which is the matrix used as input to ADELLE.

### Test statistics included in the comparison

We tested the type 1 error rate and power of ADELLE as well as the following methods for testing the global null hypothesis. For each replicate a vector of Z scores was generated as described above and given as input to each method.

#### Min

p For each Z score vector, we found the maximum of the absolute values of the Z scores and computed the two-sided p-value. The test statistic for the vector is this p-value multiplied by D. That is, we Bonferroni corrected the p-value for the number of traits tested and used the result as the test statistic for the replicate.

#### Simes

We obtained the two-sided p-values for each element in the simulated Z score vector and corrected each according to Simes’s method [16]. That is, the p-values are sort to obtain $p_{\left(1\right)}\leq p_{\left(2\right)}\leq \cdots \leq p_{\left(D\right)}$. The test statistic is ${min}_{i}\frac{p_{\left(i\right)}D}{i}$.

#### Sum of $Z^{2}$

The test statistic is $\sum_{i}Z_{i}^{2}$ the sum of the squares of the Z scores. Under independence of the Z scores, this would be $\chi^{2}$ distributed. Although we can compute an approximate distribution under correlation using analytical methods [37–39], here, we generate an empirical null distribution through simulation, as described above.

#### CPMA

We used our own implementation of the method described in [14] to compute the CPMA statistic. We computed a two-sided p-value for each Z score in the Z score vector and performed a likelihood ratio test where under the null the vector of the log of the p-values is distributed as an exponential distribution with rate equal to one and under the alternative is distributed as an exponential distribution with rate λ. Because this does not account for the correlations in the p-values we computed significance of the chi-squared likelihood ratio statistic from the empiric null distribution as described above.

In addition we considered both the GHC [25] and GBJ [26] methods but were unable to successfully run the available software on the scale of problems we consider here.

## Results

### Power and type 1 error

We tested both type 1 error and power at a significance level of α=0.01. As seen in Table 1, all methods control the type 1 error rate at the nominal level when significance is evaluated using the Monte Carlo approach.

As seen in Table 2, ADELLE has the highest power for alternatives in which the number of associated traits is 20 or larger, out of 10,000 traits. When the number of associated traits gets smaller (i.e. R=10), Simes’s method becomes the most powerful. Indeed, Simes’s method, as expected, always dominates the Bonferroni corrected min-p statistic, though the difference is small. Also, as expected, the $\sum Z^{2}$ statistic does increasingly well as the number of alternatives increases. The use of an empirical null distribution could have the effect of slightly boosting the power of Min p and Simes in our simulations, compared to what would be obtained in practice, because the usual assessment of significance for these methods is known to be slightly conservative. However, we expect this effect to be small.

### Trans eQTLs in an advanced intercross line

Gonzales et al. [4] described an advanced intercross line (AIL) of mice and undertook GWAS and eQTL mapping studies in this population. They report finding thousands of cis and trans eQTLs across three brain regions. Here, we focus on trans eQTL associations in the hippocampus region and use summary statistics to test for trans eQTL associations that were not significant in the original study. Details of the data set and original analysis can be found in Gonzales et al. [4].

For expression traits in the hippocampus, Gonzales et al. determined that in their dataset a p-value threshold of 9.01 × 10^−6^ corresponded to genome-wide significance of 0.05 when correcting for SNP-wise multiple testing, based on a permutation analysis. This value of 9.01 × 10^−6^ would thus be an appropriate significance threshold for testing a single trait with M SNPs across the genome, and it would also be an appropriate threshold for a global testing method such as ADELLE, Simes or CPMA, in which the p-values for a given SNP with each possible trait are combined into a single test statistic, resulting in M tests performed. However, if one instead takes a non-global-testing strategy of considering all the p-values for every possible pairing of a SNP and one of its trans traits, then in order to identify a SNP as a trans eQTL with a type 1 error rate of 0.05, it is necessary to correct for both the number of SNPs and the number of traits tested. For any SNP in this study there are approximately 15,000 trans genes against which it is tested. After doing a Bonferroni correction, we, therefore, consider a single SNP-trans gene association to be statistically significant if its p-value is less than 6.4 × 10^−10^.

In the supplementary information to their article, Gonzales et al. list all trans associations (where a “trans association” is defined to be any association signal that is detected between a SNP and an expression trait for a gene where the SNP and the gene are located on different chromosomes) in the hippocampus that had p-value less than 9.01 × 10^−6^, which corresponds to the threshold when correcting for M tests. Thus, many of the listed potential trans eQTLs do not meet the more stringent significance level of 6.4 × 10^−10^ required when correcting for both SNP-wise and trait-wise multiple testing.. A number of SNPs, however, particularly on chromosome 12, show evidence of some association (i.e. they have significance level between 9.01 × 10^−6^ and 6.4 × 10^−10^) with at least one trait. SNPs such as these, which show a sub-significant level of association across multiple genes, are missed when using a statistic such as min-p with a Bonferroni correction. We, therefore, chose to reanalyze the data using ADELLE and, in particular, focus on this area of chromosome 12.

ADELLE only requires summary statistics, but the available results for this data set only include summary statistics for associations that had p-value less than 9.01 × 10^−6^. We, therefore, regenerated the complete set of SNP-gene expression Z scores. We downloaded the G50–56 LGxSM AIL GWAS data set available at https://palmerlab.org, filtered the genotype dosage file to include only those mice that had gene expression data in the hippocampus, and pruned SNPs that were in complete LD using Plink. We used the downloaded genotype expression matrix for the hippocampus that had all covariates regressed out and was quantile normalized. Following the code provided in the supplementary information of Gonzales et al., we used the software package Gemma to construct LOCO GRMs and to do association analysis between each SNP-gene expression pair. We extracted Z scores from the resulting output and applied our eigenvalue shrinkage method to the traitwise Z score correlation matrix, as described above. Using the Monte Carlo assessment of significance based on 10^7^ replicates, we determined that the ELL statistic value of 3 × 10^−20^ corresponded to the genomewide significance cutoff of 9.01 × 10^−6^ that is needed to correct for SNP-wise multiple testing in this dataset.

**Fig 1.**
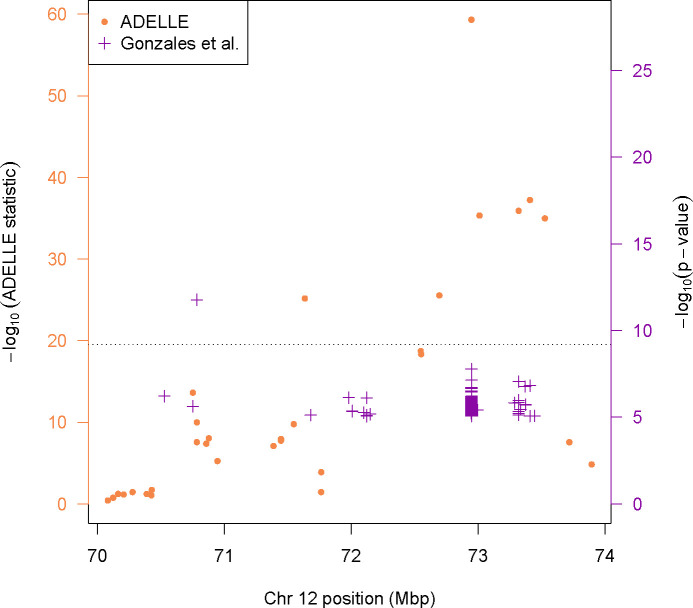
Trans eQTL associations. The region on chromosome 12 showing novel trans eQTLs. The left hand axis is for the orange points and shows the −log_10_ of the ADELLE statistic for each SNP. The right axis is for the purple points and shows the −log_10_ of the SNP-gene expression association p-values reported by Gonzales et al. Only p-values less than 9.01 × 10^−6^ were reported. The axes are scaled to have genomewide significance for a SNP (following Bonferroni correction for the number of traits tested for the purple points) be the dotted line.

In Fig 1 we can see the results of our reanalysis along with the reported results of Gonzales et al. The purple “+” symbols in the figure represent single SNP-trait associations in the Gonzales et al. analysis that had p-value less than 9.01 × 10^−6^. The −log_10_ of these p-values are displayed on the right-hand axis. The ADELLE analysis result for each SNP in the region is shown as an orange dot with corresponding scale on the left-hand axis. The axes of both sets of points are scaled to have the same dotted line as the genomewide significance threshold to declare a SNP a statistically significant trans eQTL. Note that this dotted line is more stringent than the one used in Gonzales et al. because we have applied a Bonferroni correction for the number of traits (i.e. gene expressions) tested at each SNP. Because Gonzales et al. may report multiple trait associations for a single SNP, a single SNP may appear multiple times with different p-values in the figure. We do see from the figure that only one SNP in this region surpasses the threshold to be a trans eQTL in the Gonzales data set. According to our analysis using ADELLE, however, several of the previously non-significant SNPs become highly significant. This is most clearly evident in the single SNP at approximately 72.9 Mbp which had many small but sub-significant associations in the Gonzales analysis but had a very highly significant statistic using ADELLE. We also see instances where there are SNPs that are significant or nearly significant using ADELLE, but that do not have any single association with a gene expression that was small enough to be reported by Gonzales et al. We do see one case where an association was significant in the Gonzales et al. analysis but did not reach significance with ADELLE. This is a case where a single trait had a very strong association but there was otherwise little deviation away from the null distribution with the other genes. This is a situation where a statistic like min-p is expected to do relatively well and underscores that there is no single uniformly most powerful method.

## Discussion

For trans-eQTL mapping, in order to meet rigorous standards of genomewide significance, the common strategy of considering the entire set of p-values for testing each SNP against each trans trait requires a severe multiple testing correction, because both SNP-wise and trait-wise correction is required. The resulting threshold is too strict for anything other than extremely strong associations to pass. Since a trans-eQTL association signal is not expected to be particularly large, this strategy does not seem well-suited to detecting trans-eQTLs. A global testing strategy in which association test statistics for a single SNP are combined across multiple expression traits into a single test statistic for each SNP has the potential help alleviate this problem because the resulting global test p-values need only be corrected for the number of SNPs. Whether a global test actually represents an improvement can depend entirely on the form of the global test. For example, the global test based on min-p which is one of the methods considered in our simulations is essentially the same as the common strategy.

We have developed a global testing method ADELLE that is tailored for trans-eQTL mapping. ADELLE is designed to have high power when a trans-eQTL is associated with multiple expression traits, where the proportion of associated traits is small as a subset of all traits tested, and where the individual effects sizes may be relatively weak. We have shown through a reanalysis of a mouse AIL data set that our method, ADELLE, is able to find trans eQTL signal that would otherwise not be detected when only individual SNP-trait p-values are considered.

In our simulations, ADELLE had much stronger power than other methods when the number of associated expression traits represented around 0.2%–2% of the total number of traits tested. This is a particularly relevant range for trans eQTLs because it is expected that they will often be associated with many, rather than just a single, gene. In fact, as seen in our analysis of the AIL, ADELLE is able to reject the global null hypothesis even when none of the individual trait p-values for a SNP are particularly small (i.e., they do not meet the significance threshold when correcting only for SNP-wise multiple testing, much less the more stringent standard of correcting for both SNP-wise and trait-wise multiple testing). This shows the ability of ADELLE to effectively combine multiple sub-significant association signals for a given SNP to enable genome-wide significant trans-eQTL detection.

ADELLE needs only summary statistics (either Z scores or else p-values and the signs of the estimated effect sizes) to perform its analysis. A distinct advantage of a method that only requires summary statistics is the ease with which they can be shared. This is especially relevant in human data where concerns regarding privacy and the risk of re-identification can make the sharing of original, individual level data problematic. In addition, sharing of summary statistics avoids the duplication of computation and effort that results when the original data must go through the process of quality control, normalization, testing, etc. multiple times. Sharing of the summary statistics is not without burden, however. The storage and sharing of summary statistics can be demanding, particularly in trans-eQTL studies where pairwise combinations of SNPs and genes result in a very large number of tests. Currently, ADELLE uses the complete set of Z scores to estimate the correlation matrix, though the global test is only based on the qD most significant results for each SNP, where q is set by the user. The complete set of Z scores, however, may not be available. If the complete set of Z scores is unavailable, an appropriate panel of gene expression data could instead be used in determining the needed correlation matrix. In addition, ADELLE could in principle be modified to use only the summary statistics for tests that meet a certain pre-specified significance level, rather than using a fixed number of top results for each SNP.

Computing the ELL statistic at a SNP is not time consuming. This is especially true when a precompute grid is used for the l-values. On a desktop computer, the precompute grid for this study took less than one minute. With this grid on the order of 1,000 SNPs can be analyzed per minute. The primary computational burden results from the Monte Carlo approach to determining the null distribution of our statistic. A more efficient approach to determine statistical significance is an area for future work.

Understanding the underlying biological mechanisms of trans acting effects on gene expression is a challenging task that will involve combining evidence from various lines of investigation. Here we focused on the statistical problem of identifying SNPs that affect variation in gene expression of distant genes. The combination of relatively weak effects with a very large number of tests make this a particularly difficult problem. The statistical methodology we developed for this problem, however, is general and can easily be applied to a larger set of common problems in genomics. Most any problem that involves an aggregating, or a set-based, test may benefit from our approach. For instance, tests of gene sets, SNP sets, and pathways fall into this category as do phenome wide association tests and tests which involve potential interactions when there are many possibly interacting variables, such as epistasis. In fact, as technology in the field of genomics progresses, and the number of variables, conditions and contexts grows with the size of data sets, we expect highly sensitive methods such as ADELLE to be a valuable tool in the process of developing deeper insights from the data.

## Supplementary Material

Supplement 1

## Figures and Tables

**Table 1.** Type 1 error rates of different global testing methods. at nominal level 0.01

Statistics	Type 1 error rate

min-p	0.0089
Simes	0.0089
$\Sigma \;Z^{2}$	0.0105
CPMA	0.0102
ELL	0.0093

Type 1 error is based on 10^4^ replicates in each case. The acceptance region for a test (at level 0.05) of whether the type 1 error rate differs from the nominal 0.01 level based on 10^4^ replicates is (0.00805, 0.01195) in each case.

**Table 2.** Power of the tested methods.

Number of Associated Traits					
Statistic	R=10	R=20	R=50	R=100	R=200

ELL	0.368 (.015)	0.706* (.014)	0.932* (.008)	0.935* (.008)	0.946* (.007)
min-p	0.441* (.016)	0.537 (.016)	0.517 (.016)	0.296 (.014)	0.181 (.012)
Simes	0.451* (.016)	0.555 (.016)	0.539 (.016)	0.303 (.015)	0.185 (.012)
$\Sigma \;Z^{2}$	0.042 (.006)	0.119 (.010)	0.379 (.015)	0.636 (.015)	0.872 (.011)
CPMA	0.030 (.005)	0.093 (.009)	0.298 (.014)	0.535 (.016)	0.816 (.012)

R denotes the true number of associated traits out of 10^4^ total traits. Power is tested at level 0.01 based on 10^3^ replicates in each case. Standard errors are in parentheses. A starred number denotes the highest power attained or power that is not significantly different from the highest power attained by any of the methods in the given setting.
