# Supplementary material for: ADELLE: A global testing method for Trans-eQTL mapping
Source: bioRxiv. 2024 Feb 26:2024.02.24.581871. Preprint. [Version 1] doi: 10.1101/2024.02.24.581871 (PMC10925110; doi:10.1101/2024.02.24.581871)
Supplement: Supplement 1 [file media-1.pdf]

ADELLE: A global testing method for Trans-eQTL mapping

S1 Text

Covariance matrix estimation

In order to estimate Ω, the covariance matrix of Z in Eq (5) in the main text, we make additional modeling assumptions. In an eQTL mapping study in which $\tilde{D}$ expression traits and M genome-wide SNPs are observed on each of N individuals, define G to be the $M \times N$ genotype matrix for the SNPs and Y to be the $\tilde{D} \times N$ phenotype matrix for the expression traits. Let Z denote the $\tilde{D} \times M$ matrix of test statistics, where Z_{dm}, the (d, m)th entry of Z, is the test statistic for association between trait d and SNP m, and where each Z_{dm} is assumed to be standard normal under the null hypothesis of no association between trait d and SNP m. We further assume that $N \ll \min(\tilde{D}, M)$, i.e., there are many more expression traits and SNPs than there are individuals in the study.

Special case: simple linear regression model

In the simplest case, suppose that there is no population structure or relatedness in the sample, and there are no confounding variables. Then under the null hypothesis that none of the SNPs are eQTLs for any of the traits, the N columns of Y are assumed to be i.i.d. draws from $N_{\tilde{D}}(\mu_{trait}, V_{trait})$, where μ_{trait} is the $\tilde{D} \times 1$ vector of trait means and V_{trait} is the $\tilde{D} \times \tilde{D}$ trait covariance matrix, and the N columns of G are assumed to be independent, each having mean vector μ_{geno} of length M and $M \times M$ covariance matrix V_{geno}. Furthermore, suppose that Z_{dm} is obtained as the t-statistic for testing significance of SNP m in a simple linear regression of trait d on SNP m, for $1 \leq d \leq \tilde{D}, 1 \leq m \leq M$. Then for reasonably large N (even when $N \ll \min(\tilde{D}, M)$), the covariance structure of the elements of Z is given by

$$\text{Cov}(\text{vec}(Z)) = C_{geno} \otimes C_{trait}, \quad (1)$$

where C_{geno} is the correlation matrix derived from V_{geno} and C_{trait} is the correlation matrix derived from V_{trait}. A consequence of this model is that each row of Z has covariance matrix C_{geno} and each column of Z has covariance matrix C_{trait}.

Since each element of Z is marginally standard normal and they have the correlation structure in Eq 1, it would be tempting to suppose that the joint distribution of the elements of Z must be multivariate normal, and to suppose that therefore Z must have the matrix normal distribution $\text{MN}_{\tilde{D},M}(\mathbf{0}, C_{trait}, C_{geno})$. However, this is false. In fact the distribution of Z is a mixture of matrix normal distributions, where each component of the mixture has mean 0 and variance 1 for every element of Z, but where different mixture components have different correlation matrices for $\text{vec}(Z)$. One way to specify the distribution of Z in this case is to note that conditional on G, Z does have a matrix normal distribution in large samples:

$$Z|G \sim \text{MN}_{\tilde{D},M}(\mathbf{0}, C_{trait}, \hat{C}_{geno}) \tag{2}$$

where $\hat{C}_{geno}$ is the $M \times M$ sample correlation matrix of the given G, which is a matrix of rank $N-1$. From this formulation, it can be seen that each Z is only of rank $N-1$, which makes sense, because there are only N observations of each phenotype and genotype on which the entire matrix Z is based. It can also be seen that the unconditional distribution of Z will be a mixture of matrix normals, where we mix over G. For simulation purposes, it is handy to note that it is also true in this case that

$$Z|Y \sim MN_{\tilde{D},M}(\mathbf{0}, \hat{C}_{trait}, C_{geno}), \tag{3}$$

where $\hat{C}_{trait}$ is the $\tilde{D} \times \tilde{D}$ sample correlation matrix of the given Y, so $\hat{C}_{trait}$ is of rank $N-1$.

In this simple setting, $\Omega = C_{trait}$, so we could directly estimate C_{trait} by first forming the sample correlation matrix $\hat{C}_{trait}$ for the $\tilde{D}$ traits. However, the estimate $\hat{C}_{trait}$ would be low rank because typically $N \ll \tilde{D}$, so we could regularize it by eigenvalue shrinkage [1], as described below in subsection **Regularization of sample covariance matrix**, to finally obtain $\hat{\Omega}$ as an estimate for Ω.

More general case

If there are important covariates, population structure or related individuals in the model for Y (where we assume these are properly accounted for in the association test), then the simple model for Z given in Eq 2 does not hold. However, motivated by the simple case, we propose the more general null model for Z:

$$Z|G \sim MN_{\tilde{D},M}(\mathbf{0}, \Omega, C_G), \tag{4}$$

where C_G is a $M \times M$ correlation matrix that is a function of G and has rank $N-1$ (or, more generally, $\text{rank}(C_G) = N-k-1$ if k PCs, PEER factors, or other covariates have been regressed out of the expression traits), and Ω is a $\tilde{D} \times \tilde{D}$ correlation matrix, where $\text{rank}(\Omega) = \text{rank}(C_{trait})$, which we assume is equal to $\tilde{D}$.

Properties of this model are that Z is of rank $N-k-1$, every column of Z has covariance matrix Ω and every row of Z has covariance matrix C_G. In this case, Ω is no longer exactly the trait correlation matrix, so we estimate it from Z instead of directly from Y.

A simple (but low-rank) estimator would be the sample correlation matrix for the rows of Z, given by $\text{cov2cor}(Z(I_M - M^{-1}\mathbf{1}_M\mathbf{1}_M^T)Z^T$, where cov2cor is the function that maps a symmetric positive semi-definite matrix A with positive diagonal elements to a matrix of the same size with (i,j)th element $A_{ij}/\sqrt{A_{ii}A_{jj}}$. If C_G were available, a more efficient (but still low-rank) estimator could be obtained by both decorrelating and centering the rows of Z to obtain $\text{cov2cor}(Z(C_G^- - C_G^-\mathbf{1}_M(\mathbf{1}_M^T C_G^-\mathbf{1}_M)^{-1}\mathbf{1}_M^T C_G^-)Z^T)$, where C_G^- is the Moore-Penrose generalized inverse of the (singular) matrix C_G. More generally, suppose we use matrix $\tilde{C}^-$ in the estimator $\hat{\Omega}_1 = \text{cov2cor}(Z\tilde{C}^- Z^T)$, where $\tilde{C}^-$ is a symmetric, positive semi-definite matrix.

To decide which choice of $\tilde{C}^-$ to use in the estimator $\hat{\Omega}_1$, we consider the following simple setting for estimation of a single correlation value ρ. Suppose $(X_1, Y_1), \ldots, (X_n, Y_n)$ are i.i.d. bivariate normal with mean vector $(0, 0)$ and with 2×2 covariance matrix having both diagonal elements equal to 1 and both off-diagonal elements equal to ρ. Then, if we let $\rho_{d1,d2}$ denote the $(d1, d2)$th entry of Ω, where $d1 \neq d2$, in large samples the $(d1, d2)$th entry of $\hat{\Omega}_1$ would be approximately unbiased for $\rho_{d1,d2}$ with variance $(1-\rho_{d1,d2}^2)^2 \text{tr}(C_G\tilde{C}^- C_G\tilde{C}^-)/[\text{tr}(C_G\tilde{C}^-)]^2$, which is minimized when $\tilde{C}^- = C_G^-$, in which case it becomes $(1-\rho_{d1,d2}^2)^2/\text{rank}(C_G)$. However, to increase the robustness of the estimator, we usually prefer to choose a $\tilde{C}^-$ that is orthogonal to 1_M, so that we are also centering Z. Within this class of estimators, the variance is minimized when $\tilde{C}^- = C_G^- - C_G^-\mathbf{1}_M(\mathbf{1}_M^T C_G^-\mathbf{1}_M)^{-1}\mathbf{1}_M^T C_G^-$, which leads to the second low-rank estimator suggested above. The resulting variance of the estimator is $(1-\rho_{d1,d2}^2)^2/(\text{rank}(C_G)-1)$.

Possibilities we have considered in practice are using $\tilde{C}^- = I - M^{-1}1_M 1_M^T$ (which makes $\hat{\Omega}_1$ the sample correlation matrix) or $\tilde{C}^- = \hat{C}_{geno}^- - \hat{C}_{geno}^-\mathbf{1}_M(\mathbf{1}_M^T\hat{C}_{geno}^-\mathbf{1}_M)^{-1}\mathbf{1}_M^T\hat{C}_{geno}^-$, where $\hat{C}_{geno}$ is the sample correlation matrix of G, which would be the optimal choice among all $\tilde{C}^-$ that are orthogonal to 1_M, in the special case of simple linear regression. In the simulations and data analysis, we have used $\tilde{C}^- = I - M^{-1}1_M 1_M^T$. Given $\hat{\Omega}_1$, which is generally of rank $N-k-1$, we apply regularization to obtain full-rank $\hat{\Omega}$ as follows.

Regularization of sample covariance matrix

To regularize $\hat{\Omega}_1$, we apply a form of eigenvalue shrinkage. Suppose $\hat{\Omega}_1 = P\Lambda P^T$ is the eigendecomposition of $\hat{\Omega}_1$, where Λ is a diagonal matrix with dth diagonal element λ_d, the dth eigenvalue, where $\lambda_1 \geq \lambda_2 \geq \ldots \lambda_D \geq 0$, and since $\hat{\Omega}_1$ has 1's on the diagonal, we also have $\sum_{d=1}^D \lambda_d = D$. As a first step, we apply shrinkage to calculate a new set of eigenvalues $\tilde{\lambda}_1 \geq \tilde{\lambda}_2 \geq \ldots \geq \tilde{\lambda}_D \geq 0$, and a new diagonal matrix $\tilde{\Lambda}$ whose dth diagonal element is $\tilde{\lambda}_d$, as follows: for our setting in which the number of observations N is small relative to D and M, we define $\gamma = \dim(\hat{\Omega}_1)/\text{rank}(\hat{\Omega}_1)$ and let $\lambda_+ = (1+\sqrt{\gamma})^2$. Let $h = |\{d : \lambda_d > \lambda_+\}|$, and call

$\{\lambda_1,\ldots,\lambda_h\}$ the "large" eigenvalues and $\{\lambda_{h+1},\ldots,\lambda_D\}$ the "small" eigenvalues. We apply a debiasing function f_1 to each of the large eigenvalues and a linear contraction f_2 to each of the small eigenvalues. Here $f_1(\lambda_d) = .5(\lambda_d + 1 - \gamma + \sqrt{(\lambda_d + 1 - \gamma)^2 - 4\lambda_d})$, where f_1 is the inverse bias function for the large eigenvalues in a spiked covariance model for a case when $\hat{\Omega}_1$ is not low-rank [2]. In that non-low-rank case, $f_2(\lambda_i)$ would ordinarily be a constant function equal to 1, but in a low-rank setting, we find that shrinkage to be too severe, so we instead use a linear contraction $f_2(\lambda_d) = a + b * \lambda_d$, where a and b are chosen to satisfy the constraints that $f_2(\lambda_+) = f_1(\lambda_+)$, i.e., that f_1 and f_2 agree at the boundary between small and large eigenvalues, and that $\sum_{d=1}^{D} \tilde{\lambda}_d = D$. The resulting a and b are given by $a = 1 + \sqrt{\gamma} - b\lambda_+$ and $b = (\sqrt{\gamma} + 1 - r)/(\lambda_+ - \bar{\lambda}_{small})$, where $r = (D - \sum_{d=1}^{h} \tilde{\lambda}_d)/(D - h)$ and $\bar{\lambda}_{small} = (D - h)^{-1} \sum_{d=h+1}^{D} \lambda_d$. Note that $a > 0$ and $0 < b < 1$. Given $\tilde{\Lambda}$, we obtain our final covariance matrix estimator $\hat{\Omega}$ as

$$\hat{\Omega} = \text{cov2cor}(P\tilde{\Lambda}P^T). \tag{5}$$

Beta-binomial approximation

In this subsection, we describe the beta-binomial approximation [3] used to calculate values of $F_{(d)}$, the cdf of $\pi_{(d)}$, for $d = 1,\ldots,qD$, which is needed for obtaining the l-values used in forming the ADELLE test statistic. From Eq (7) in the main text, we have for the l-value

$$l_d(h) \equiv F_{(d)}(h) \equiv P_0(\pi_{(d)} \leq h) = P_0(S(-\Phi^{-1}(h/2)) \geq d), \tag{6}$$

where $S(c) = \sum_{d=1}^{D} \mathcal{I}\{|Z_d| \geq c\}$. If $\Omega = I$, then for $c \geq 0$, $S(c)$ has the null distribution of a Binomial$(D, 2\Phi(-c))$ random variable, and

$$F_{(d)}(h) = 1 - \sum_{j=0}^{d-1} \binom{D}{j} h^j (1-h)^{D-j}, \quad h \in [0,1], \tag{7}$$

which is the CDF of a Beta$(d, D+1-d)$ distribution. When $\Omega \neq I$, the null mean of $S(c)$ will remain the same, $E_0[S(c)] = 2D\Phi(-c)$, however the null variance is

$$\begin{aligned} V_0(S(c)) &= V_0\left(\sum_k \mathcal{I}\{|Z_k| \geq c\}\right) \\ &= 2D\Phi(c)(1-2\Phi(c)) + 2\sum_{i=1}^{D-1}\sum_{j=i+1}^{D} \text{cov}\left(\mathcal{I}\{|Z_i| \geq c\},\ \mathcal{I}\{|Z_j| \geq c\}\right) \\ &> 2D\Phi(c)(1-2\Phi(c)) \end{aligned}$$

Where the last inequality follows from the positive correlation of the magnitudes of any correlated, mean-zero, bivariate Gaussians.

Following the method proposed by [3] (see also [4]), we approximate the distribution of $S(c)$ with a beta-binomial distribution $BB(D, a, b)$. The beta-binomial is the distribution formed from the binomial distribution when the success probability is drawn from a beta distribution. It is convenient to reparameterize the beta-binomial in terms of parameters $\lambda = \frac{a}{a+b}$ and $\gamma = \frac{1}{a+b}$. If X is drawn from a BB($D,\ \lambda,\ \gamma$), where $\lambda > 0$, $\gamma > 0$, and D is a positive integer, then X has the following properties:

$$f_{D,\lambda,\gamma}(x) \equiv P(X = x) = \binom{D}{x} \frac{B(\frac{\lambda}{\gamma} + x, \frac{1-\lambda}{\gamma} + D - x)}{B(\frac{\lambda}{\gamma}, \frac{1-\lambda}{\gamma})}, \tag{8}$$

$$\lambda = E(X)/D, \text{ and} \tag{9}$$

$$\frac{\gamma}{1+\gamma} = \frac{V(X) - D\lambda(1-\lambda)}{D(D-1)\lambda(1-\lambda)}, \tag{10}$$

where $B(a, b)$ is the beta function.

To approximate the distribution of $S(c)$ we choose λ and γ by the method of moments. Barnett et al. [3] derived the following:

$$\begin{aligned} E_0\left[S\left(c\right)\right] &= 2D\Phi(-c) \\ V_0\left[S\left(c\right)\right] &= D\left[2\overline{\Phi}(c) - 4\overline{\Phi}^2(c)\right] + 4D(D-1)\phi^2(c)\textstyle\sum_{i=1}^{\infty}\mathcal{H}_{2i-1}^2(c)\rho(2i)/(2i)! \end{aligned} \tag{11}$$

Where $\mathcal{H}_i$ are the Hermite polynomials, $\rho(i) = \frac{2}{D(D-1)}\sum_{1\leq k<l\leq D}\left(\Omega_{kl}\right)^i$, where Ω_{kl} is the (k, l)th entry of Ω, and ϕ and $\overline{\Phi}$ are the density and survivor functions of a standard normal, respectively. Note that Eqs 9, 10, and 11 imply that once λ is set by the method of moments, knowing λ determines c which determines $V_0\left[S\left(c\right)\right]$ and thus determines γ. So we will drop the writing of γ (and D) below to simplify notation, and define $f_\lambda(x)$ to be the beta-binomial density with parameters (D, λ, γ), where λ and γ are related by the equations $\lambda = 2\Phi(-c)$ and

$$\frac{\gamma}{1+\gamma} = \frac{V_0[S(c)] - D\lambda(1-\lambda)}{D(D-1)\lambda(1-\lambda)},$$

with $V_0[S(c)]$ given in 11.

Applying this to Eq 6,

$$\begin{aligned}
l_d(h) \equiv F_{(d)}(h) &= P_0\left[\pi_{(d)} \leq h\right] \\
&= P_0\left[S\left(-\Phi^{-1}\left(h/2\right)\right) \geq d\right] \\
&= 1 - \sum_{k=0}^{d-1} P_0\left[S\left(-\Phi^{-1}\left(h/2\right)\right) = k\right] \\
&\approx 1 - \sum_{k=0}^{d-1} f_h\left(k\right) \\
&= \sum_{k=d}^{D} \binom{D}{k} \frac{B(\frac{\lambda}{\gamma}+k, \frac{1-\lambda}{\gamma}+D-k)}{B(\frac{\lambda}{\gamma}, \frac{1-\lambda}{\gamma})} \\
&\equiv \hat{F}_{(d)}(h) \equiv \hat{l}_d(h).
\end{aligned} \tag{12}$$

Pre-computation for the ADELLE test

Our ADELLE method lends itself to a pre-computation to reduce computation time when it will be applied to a large number of SNPs and a large number of traits. Suppose we observe M SNPs along with the D traits. Then, from Equation (12), calculating M ADELLE statistics involves $M \times \sum_{j=1}^{qD}(D-j+1) \approx MqD^2$ evaluations of a beta-binomial probability mass function $f_h(k)$. However, we also see that computation of $\hat{l}_1(h)$ necessarily also computes all the l-values $\hat{l}_1(h), \ldots, \hat{l}_D(h)$.

This motivates a computationally efficient strategy for approximating the function $\hat{F}_{(d)}(h)$ for arbitrary h and $1 \leq d \leq qD$ by pre-computing a $qD \times H$ matrix of l-values, where H is the number of values of h in the grid. We choose a set of pre-computation points, $\mathcal{H} = \{h_1, \ldots, h_H\}$, where $0 < h_1 < \cdots < h_H = 1$, and evaluate each $\hat{l}_d$ only on the points in $\mathcal{H}$. For $1 \leq d \leq qD$ but $h \notin \mathcal{H}$ we linearly interpolate when $h_1 < h < h_H$,

$$\hat{l}_d(h) \approx \hat{l}_d(h_i) + \frac{\hat{l}_d(h_{i+1}) - \hat{l}_d(h_i)}{h_{i+1} - h_i}(h - h_i)$$

where $h_i < h < h_{i+1}$. For $h < h_1$ we compute the l-value directly from (12).

In general both the computation time and the accuracy of the approximation tend to increase with the number of points H and the density of the pre-compute grid. In the data analysis, we use $H = 10^5$ and $h_1 = 10^{-20}$. A naive means of assigning the values of $\mathcal{H}$ would be to create an evenly spaced grid between h_1 and 1, but this devotes few points, and little resolution to small values of h. Instead we choose $\mathcal{H}$ to be the geometric sequence $h_i = h_1^{\frac{H-i}{H-1}}$ for $1 \leq i \leq H$. This results in 5,000 grid points for each order of magnitude, scaled logarithmically, for our choice of h_1 and H.

References

1. Johnstone IM, Paul D. PCA in High Dimensions: An Orientation. Proc IEEE. 2018;106(8):1277–1292. doi:10.1109/JPROC.2018.2846730.

2. Donoho D, Gavish M, Johnstone I. Optimal shrinkage of eigenvalues in the spiked covariance model. Ann Stat. 2018;46(4):1742–1778. doi:10.1214/17-AOS1601.

3. Barnett I, Mukherjee R, Lin X. The generalized higher criticism for testing SNP-set effects in genetic association studies. J Am Stat Assoc. 2017;112(517):64–76. doi:10.1080/01621459.2016.1192039.

4. Sun R, Lin X. Genetic variant set-based tests using the generalized Berk–Jones statistic with application to a genome-wide association study of breast cancer. J Am Stat Assoc. 2020;115(531):1079–1091. doi:10.1080/01621459.2019.1660170.
